# A Study of the Tennis Trust-Based System on the Psychology and Cognition of Amateur Tennis Enthusiasts

**DOI:** 10.3390/bs14111019

**Published:** 2024-11-01

**Authors:** Fanghuan Yang, Yi-Sub Kwak

**Affiliations:** DEU Exe-Physio Lab, Department of Physical Education, Dong-Eui University, 176, Eomgwangno, Busanjin-gu, Busan 47340, Republic of Korea

**Keywords:** amateur tennis, trust-based, the grounded theory, the perceptual cycle model (PCM), psychology

## Abstract

Currently, amateur tennis events are developing rapidly, and the trust system has become a common rule, integrating considerations of entertainment, fitness, and cost control. However, human-centered trust system rules still face some controversy, and there is limited specialized research on the topic. This study explores amateur tennis players’ perceptions of the trust system rules. Using focus group discussions and interviews, we collected semi-structured interview data from 23 participants in tennis events. Based on grounded theory and the perceptual cycle model (PCM) framework, we developed a theoretical model of the tennis trust system and a model of the operational mechanism of the tennis trust system. Based on the grounded theory model results, four main factors influencing the tennis trust system were identified: interest orientation, information acquisition and judgment, communication and interaction, and development strategies. The operational mechanism model based on the PCM framework explains that the functioning of the tennis trust system includes five stages: foundation stage, trust-based emergence stage, monitor and detect stage, anticipate and respond stage, and development improvement stage. Among these stages, the anticipate and respond stage is crucial for the effectiveness of the trust system and is also the stage most prone to controversy. To address this, we propose targeted improvements to enhance the fairness of the tennis trust system and meet the needs of amateur tennis events.

## 1. Introduction

Since its inception in the late 19th century, tennis has rapidly gained worldwide popularity, becoming a significant event in important international competitions such as the Olympics and the four Grand Slam tournaments. This has greatly contributed to the popularization and development of the sport [1]. In recent years, with the rise of the national fitness movement and the increasing awareness of health, amateur tennis competitions have gradually emerged both domestically and internationally, attracting a large number of tennis enthusiasts to participate [2,3,4]. In China, tennis tournaments have gradually evolved from being considered “elite events” to “white-collar events” and are now increasingly becoming “popular events”. The number of cities hosting tennis tournaments is steadily increasing, the scale of the events is expanding, the number of participants is rising, and the variety and categories of events are continuously diversifying [5]. Starting in 2009, the Beijing CRT China Open-level tournament has divided China into six regions, with each region selecting seven cities to host the competitions. This approach has not only promoted the development of local amateur tennis populations but also benefited the publicity of the events, thereby enhancing their visibility and influence [6]. According to the “2021 Global Tennis Report” published by the International Tennis Federation (ITF), approximately 20 million people in China participated in tennis (playing at least once a year) in 2020, making it the country with the second-highest number of tennis participants in the world [7]. However, ensuring fairness in these competitions remains an urgent issue that needs to be addressed.

In tennis matches, referees play a crucial role in ensuring fair play and making decisions based on the rules. One key aspect of their role is making calls on whether the ball is in or out, which is also the area most prone to disputes. At critical moments in a match, such as during an advantage, deuce, or match point, an incorrect call can result in losing a point, a game, a set, or even the entire match [8]. Such errors can also affect a player’s performance [9]. However, in actual matches, referees are prone to making incorrect calls due to the inherent limitations of their cognitive and sensory abilities, as well as various potential biases that may influence their judgment [10]. Research indicates that sports referees’ decisions are influenced by their individual prior knowledge and values, early experiences, training, and cognitive factors [11]. In summary, the accuracy of decisions in a match is considered crucial for the integrity of the sport. However, in many cases, human-driven judgments do not always provide reliable results [12]. Some scandals involving human referees and other negative influences have threatened the legitimacy of human officiating in sports. Although existing studies have provided interpretations of referees’ decision-making in officiating, these studies primarily focus on how various factors affect the accuracy of referees’ decisions [13,14]. However, there is still a lack of in-depth and systematic exploration regarding the core issue of how referees make decisions to ensure the fairness of competitions. The decision-making mechanisms of referees remain in a ‘cognitive black box’, and public doubts and controversies about the fairness of referees’ judgments remain unresolved.

To overcome issues of subjectivity and bias in refereeing and to improve objectivity and accuracy, electronic systems assisting referees have been widely adopted in competitive sports since the mid-1990s. Currently, the application of artificial intelligence technology in the field of sports refereeing has demonstrated immense potential for development, particularly in terms of revolutionizing the precision of rule enforcement and decision-making in competitive sports. In tennis, the introduction of the challenge system using artificial intelligence began in 2006 [15]. Artificial intelligence, in the form of video assistant referee (VAR), is used in tennis just as it is in other ball sports [16]. Among these technologies, Hawk-Eye [17] has achieved an accuracy of 2.6 mm [18]. By combining this with AI software designed for high-speed sports like badminton, it can be effectively applied to tennis [19]. Such systems have the potential to bypass the limitations of human perception and cognition by combining artificial intelligence technologies to support (or even replace) human judgment. This can help counteract or eliminate biases in officiating [20]. From the spectators’ perspective, using various electronic systems to reduce the impact of human factors on officiating can enhance the quality of the matches, make the game easier to understand, and provide real-time feedback that adds excitement for the audience [21]. The development and advancement of any technology have their dual nature. The integration of artificial intelligence into sports officiating not only receives praise for promoting fairness in sports and enhancing the accuracy of officiating but also sparks considerable controversy. Some even argue that intelligent officiating systems, such as VAR, are the ‘killers’ of sports enthusiasm [14,22]. The precision of AI has eliminated redundancies in rules, which has led to increasing rigidity of the rules themselves, becoming a point of controversy and a challenge to resolve. With AI intervention, aspects that were previously imperceptible to the human eye are now measured with millimeter-level accuracy, leaving no substantive gap between the rules and practical standards. This has resulted in a rise in calls such as fouls, invalid goals, and out-of-bounds plays. These changes have sparked discussions about the purpose of rules and prompted reflection on whether we can accept such transformations [23].

This controversy is particularly evident in professional events; however, in amateur competitions, the issues become even more complex. Most tennis events currently cater to amateur players [24]. In amateur tournaments, especially in terms of officiating and dispute resolution, the lack of professional referees and high-tech equipment means that decisions often rely on the participants’ self-judgment and negotiation. This raises a critical question: How can fairness in officiating and the smooth progression of matches be ensured in amateur competitions that lack technological tools like artificial intelligence? At this point, the role of trust in sports becomes evident. The conduct of sports activities is built on trust, which is a universally present characteristic, particularly between athletes and between athletes and coaches [25]. In competitive sports, trust is an indispensable element, influenced by three main factors: interdependence, risk, and uncertainty. Trust is prevalent between athletes, between athletes and coaches, between captains and team leaders, and between teams. In the process of sports training, interpersonal trust is far more important than competition against each other [26]. In this context, tennis matches often use a system known as the “trust-based system”, where each player acts as a referee for the opponent. In cases where the judgment of ball placement is inaccurate or ambiguous, the decision is based on the receiving player’s opinion. Therefore, for some unclear calls, the verbal statements of the players become the basis for the ruling [27]. The core principle of the trust-based system is based on mutual trust and respect among participants, aiming to ensure fairness and justice in the match through self-management and self-discipline [28]. However, timely, accurate, and reliable decisions require more experienced officials [29]. Research shows that under time pressure, the combination of situational priors and dynamic environmental background information jointly influences athletes’ judgment and decision-making [30]. Although the trust-based system has many advantages in amateur tennis tournaments, it also faces some issues and challenges in practice. Some participants may act dishonestly due to match pressure or competitive psychology, which can affect the fairness and integrity of the competition. Without a referee, the trust-based system lacks an effective dispute-resolution mechanism, which can lead to prolonged arguments and dissatisfaction during the match [31]. In summary, the trust-based system is more suitable for informal matches such as friendly games and practice sessions. Its effectiveness and feasibility in highly competitive and influential matches remain to be further validated.

Given the potential application value of the trust-based system in amateur tennis events, this study aims to explore a preliminary theoretical model. The study is based on qualitative research using grounded theory. Additionally, it addresses the issues encountered in practical operations. The goal is to provide more information on the scientific and effective application of the trust-based system in amateur tennis tournaments.

## 2. Methodology

### 2.1. Model Theory

#### 2.1.1. Grounded Theory

The grounded theory is a qualitative research method proposed by Glaser and Strauss in 1967. It involves the systematic collection and analysis of data to generate theory. The central idea is to allow the theory to be grounded in the data [32]. Unlike traditional research methods, the grounded theory emphasizes deriving theories from actual data rather than starting with a theory and then verifying it with data. During the research process, researchers conduct detailed analysis through constant comparison and open coding, identifying initial concepts and filtering out core concepts from a large amount of data, ultimately constructing a theory [33]. The study aims to explore the mechanisms and future development directions of the trust-based system in tennis based on the grounded theory. It divides the coding process into three levels: open coding, axial coding, and selective coding. Open coding requires researchers to approach the collected raw data without any subjective bias or theoretical preconceptions and, with an open mind, conduct preliminary coding to identify concepts and categories that reflect social phenomena in the investigation [34]. Axial coding, through inductive and deductive analysis, involves constant comparative analysis to deeply explore relationships between categories, leading to more abstract insights and identifying core categories [35]. Selective coding is the final phase of data analysis, where the relationships between core categories and main categories are determined through integration and refinement, thus organizing the storyline between core categories and constructing the theoretical framework [36].

#### 2.1.2. Perceptual Cycle Model

The perceptual cycle model (PCM) is a cognitive model developed by Ulric Neisser in 1976 to explain how perception, action, and the environment interact dynamically in human cognition [37]. The model illustrates how individuals actively seek information from their environment to guide their actions and how these actions, in turn, influence their perception. The PCM emphasizes the cyclical and continuous nature of this process, where perception and action are interdependent and constantly influence each other. The PCM emphasizes the dynamic interaction between perception, action, and the environment, which is closely related to the decision-making mechanisms of amateur tennis referees in complex and uncertain match situations. Referees’ decisions not only rely on current perceptual information but also involve drawing on past experiences and anticipating future situations. These characteristics align well with the research objective, which is to explore how referees make quick judgments and responses through a trust-based system during matches.

#### 2.1.3. Theoretical Framework

This study combines grounded theory and the PCM to explore the operational mechanisms of the trust-based refereeing system in amateur tennis matches. The trust-based refereeing system refers to situations where, in the case of ambiguous ball placement, the receiving player’s judgment becomes the final ruling. The purpose of this research is to reveal how athletes make decisions based on mutual trust within this system.

Through the continuous comparative analysis of grounded theory, this study identifies the core factors of trust from athletes’ actual match data, uncovering how they establish, maintain, and adjust trust in their opponents throughout the game. Meanwhile, the perceptual cycle model provides a cognitive framework for explaining the dynamic nature of trust, showing how athletes make quick judgments and take action based on environmental perception, past experiences, and trust in their opponents in ever-changing match situations.

**Figure 1:**
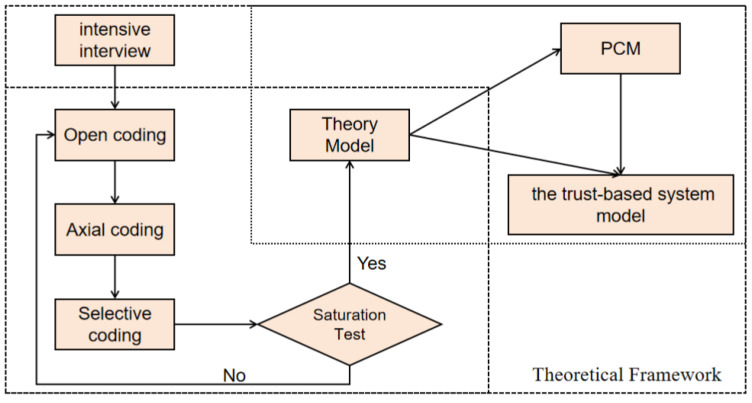
Theoretical framework.

The grounded theory and the PCM model form a complementary relationship in explaining trust-based systems (Figure 1). Grounded theory employs a bottom-up data analysis approach to reveal how participants build trust through interaction and negotiation during competitions. In contrast, the PCM model offers a cognitive-level explanation, demonstrating how participants adapt to the dynamic nature of the competition through continuous perception, feedback, and response. While grounded theory emphasizes the induction of behaviors and experiences, the PCM model supplements this by elucidating the cognitive mechanisms underlying those behaviors, helping to explain how participants make judgments, adjustments, and responses within a trust framework. In this refereeing system, trust is not just a static judgment criterion; it is a dynamic process that evolves throughout the game, influenced by multiple factors such as the match stage, score differences, and interactions between athletes. By integrating these two theories, this study not only reveals the crucial role of trust in ambiguous officiating decisions but also explores how this trust-based refereeing system impacts the fairness of amateur tennis matches and athletes’ behavioral responses.

### 2.2. Participants

The essence of the grounded theory research model is that theory is directly extracted from actual data rather than relying on a priori assumptions. This method typically employs semi-structured interviews using sampling techniques, and there is no strict requirement for the sample size. However, research indicates that a sample size of 20 to 30 interviewees is generally reasonable [38]. Therefore, based on the research objective of the tennis trust-based system, this study employed purposive sampling and randomly selected 23 amateur tennis enthusiasts from various tennis event venues in Fuzhou, China, for interviews and focus group discussions. The participants included a diverse group of individuals, including members from various industries’ elite groups. When discussing their socioeconomic status and its impact on their sports participation, opportunities, and experiences, the athletes expressed different viewpoints. Among them, the average age was 45.6 years, encompassing a variety of professions. Specific details are available in Table 1. Each participant signed an informed consent form and received a CNY 10 reward.

### 2.3. Interview Design and Data Collection

To ensure the validity of the survey results, preliminary interviews were conducted before formal interviews, and the interview outline was modified based on the effectiveness of these preliminary discussions. During the formal interviews, all participants underwent concentrated semi-structured interviews, which relied on open-ended questions as a technique, emphasizing participants’ experiences and their understanding of the tennis trust-based system [39]. Each participant’s interview lasted between half an hour and one hour, totaling 23 interviews conducted. Additionally, considering the iterative nature of the coding process in the grounded theory, further exploration was conducted in subsequent stages to understand participants’ social status and other background factors. This included aspects such as participants’ professions, family perspectives on tennis, and phenomena related to parent–child tennis activities. The final interview outline can be found in Table 2.

We conducted semi-structured 1-on-1 interviews, recording all interview conversations through audio and transcribing them verbatim into textual materials. The final textual materials were uploaded into NVivo 13 software for analysis and coding [40].

### 2.4. Model Development

We employed a constructivist grounded theory approach and utilized multiple analytical tools during the model development process [41]. Firstly, we conducted an in-depth analysis of the 23 interviews using primary coding and focused coding. Primary coding was used to explore concise and direct initial concepts, while focused coding further deepened the theory by comparing and contrasting differences between various codes and identifying directions for further exploration. The constant comparison technique was applied throughout the coding process. This technique helped identify similarities and differences between data codes. It included comparisons among participants as well as between the researchers’ and participants’ viewpoints [42].

Secondly, theoretical sampling was used to support the development of the theory. This method does not aim to increase the number of participants but rather to refine and develop theoretical concepts through iterative interviews and a literature review, redirecting, developing, and refining theoretical concepts [43]. In addition, memos played a crucial role in the category establishment and concept extraction stages, helping to describe the characteristics and conditions of categories and allowing for comparisons between categories. The use of theoretical sampling detailed and refined the developing categories, explored new theoretical interpretations through further interviews, and ultimately formed coherent theoretical models. For example, in this study, concerns about the tennis trust-based system were reported by participants. At this stage, theoretical sampling could be employed to select participants who have expressed controversies related to the trust-based system for inclusion in the investigation. These methods together form a flexible research framework that emphasizes deep theoretical development and interaction between participants and researchers [44].

Finally, using the three-level coding process of the grounded theory, we analyzed the logical relationships between primary categories and core categories. Through saturation testing (checking for the presence of new concepts or categories), we constructed the initial theoretical model of the tennis trust-based system, represented the developed theory using diagrams and charts [44]. Based on the coding derived from the grounded theory, we used the PCM to construct a trust mechanism model, explaining the reciprocal relationship between perception and action in the trust-based system. This approach highlights how humans actively construct their understanding of the world through continuous interaction with their environment.

## 3. Results

In the analysis of the results, we focused on the theoretical model constructed using the three-level coding of the grounded theory. Additionally, we developed a stage model based on the operational process of the tennis trust-based system rules to illustrate the influencing factors and mechanisms of operation of these rules.

### 3.1. Encoding Results

We systematically analyzed the interview transcripts to summarize new concepts and categories related to the trust-based system in tennis, using participants’ responses as the basis for open coding. For instance, one participant’s statement, “I have loved watching tennis matches since I was a child; tennis has always been my favorite sport, and I play almost every week”, reflects a long-standing passion for tennis and ongoing engagement with the sport as a hobby. Therefore, this response was categorized as a passion for tennis. By repeating this process, we identified a total of 28 concepts through open coding. We conducted an in-depth analysis of the differences and similarities among these concepts, restructured initial concepts, and developed relationships between concepts. This process yielded eight axial codes representing secondary categories. Building upon the secondary concepts, this study conducted an internal analysis of the eight core concepts, refining them into four primary core categories that reflect the characteristics of the tennis trust-based system.

The development of the tennis trust-based system was primarily characterized by interest-driven, information acquisition and judgment, interactive communication, and development strategy. Interest-driven formed the fundamental premise for amateur tennis enthusiasts adopting the trust-based system, involving the enjoyment derived from recreational hobbies and competitive entertainment. The assessment of subjective and objective information in tennis competitions constituted the core content of the trust-based system, specifically in terms of information acquisition and judgment. This directly influenced the rationality and contentiousness of rule application, while interactive communication involved feedback and acceptance, encompassing psychological aspects and stress. The introduction of the trust-based system concept was a form of communication between opponents in competitive matches characterized by psychological and social dimensions.

Finally, we summarized the strategies for the development of the trust-based system, highlighting its advantages, such as cost reduction and efficiency improvement. However, we also acknowledged its potential for controversy and identified certain limitations. To validate the coherence of the grounded theory model results, we conducted theoretical saturation testing on 3 out of the 23 transcripts. No new concepts or categories were identified, confirming the model’s inclusiveness and enhancing its credibility. The final conceptual model is presented in Table 3.

### 3.2. A Mechanistic Model of the Trust-Based System

**Figure 2:**
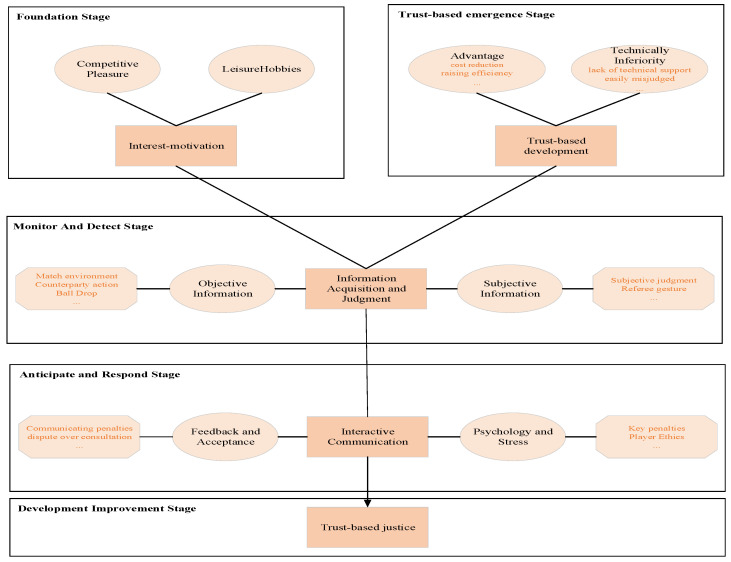
A mechanistic model of the trust-based system.

Based on the coding results from grounded theory and integrating the PCM perception–action process, we constructed a tennis trust-based system model. This model outlined the understanding mechanism of the tennis trust-based system across five stages (Figure 2), including the foundation stage, trust-based emergence stage, monitor and detect stage, anticipate and respond stage, and development improvement stage.

#### 3.2.1. Foundation Stage

The rise of any sports event is closely tied to its professionalization, which attracts more amateur enthusiasts to participate in the sport [45]. The emergence of the trust-based system in tennis indeed addresses the challenges of officiating in amateur events. It relies on the collective agreement of a sufficient number of participants to gain recognition for these new rules. This principle mirrors the collaborative spirit seen in various community-driven projects where consensus and widespread participation are crucial for success, much like your approach to involving diverse perspectives in your projects. In focus groups and discussions, participants have expressed their interest in tennis, highlighting how their passion drives them to participate in amateur events. This interest-driven forms a solid foundation for the emergence of the tennis trust-based system rules in competitive settings.

Interest-driven primarily encompasses two aspects: recreational hobbies and competitive entertainment. At the recreational hobbies level, tennis is seen as an activity that promotes health and social interaction. Participants typically engage in it to enjoy the pleasure of the game and to relax both mentally and physically. During this stage, players cultivate a fundamental understanding of tennis rules and skills through informal matches and friendly competition. Simultaneously, they build confidence in their abilities and trust in fair competition among others. At the competitive entertainment level, while participants still prioritize enjoyment, the competitive nature of the matches and the demand for skill gradually increase. During this stage, players begin participating in club tournaments and local events, aiming to enhance their technical skills and mental resilience through more challenging competitions. They experience a sense of achievement and team spirit within the competitive environment. Some participants expressed, “*I often follow professional tennis tournaments such as Grand Slams, ATP, and WTA tours. By watching top players’ matches, I can learn their techniques and tactics, gaining deeper insights into the strategies and mental battles behind the game. This not only helps me improve my own game level but also increases my passion and dedication to the sport.*” (P1). The development of professional tournaments has sparked enthusiasm among amateur players. Similarly, the evolution of amateur competition has also heightened everyone’s interest in tennis. “*Participating in tournaments is a significant challenge and opportunity for me. Each match not only tests my skills and strategies but also allows me to continually improve in competition. Regardless of the outcome, every experience brings a sense of achievement and satisfaction, motivating me to love and dedicate myself more to this sport.*” (P3).

Therefore, tennis, as both a recreational hobby and a competitive sport, forms the foundation stage of the development of the tennis trust-based system. This lays a solid groundwork for further promoting and advancing the sport.

#### 3.2.2. Trust-Based Emergence Stage

The trust-based emergence stage primarily explains the surge in amateur tennis events due to the increasing number of tennis enthusiasts. However, due to factors such as costs and facility technology, advanced AI technologies like Hawk-Eye used in professional tournaments may not be available. This could lead to contested calls, potentially influencing match outcomes. We analyzed the advantages and some disadvantages of the emergence of the trust-based system at this stage.

The emergence of the tennis trust-based system can reduce competition costs and improve efficiency, thereby addressing, to some extent, the adjudication issues in most non-professional tennis events. Amateur players discussed the advantages of the trust-based system in tennis matches, which are crucial for promoting this rule and even the sport itself. “*In many amateur matches, we rely on self-regulation and integrity to abide by the rules, which not only simplifies the adjudication process but also strengthens mutual trust.*” (P11). With the gradual implementation of the trust-based system in tennis matches, players have experienced a more challenging way of competing. This model not only promotes the development of tennis but also provides new ideas and insights for other sports. Through innovative practices in trust-based tennis competition models, it is possible to explore and discover more innovative ways to further enrich and improve competition formats. “*I believe that the rules of the tennis trust-based system not only ensure fair competition and sportsmanship but also provide opportunities to explore innovative ways. Through self-regulation and fair play, we can simplify management procedures and improve the efficiency of competitions. At the same time, this trust-based system encourages us to try new strategies and techniques during matches, promoting continuous progress and innovation both individually and across the sport.*” (P16).

It is worth noting that despite the many benefits of the tennis trust-based system for amateur events, it also faces certain issues, such as ambiguous rules, susceptibility to errors in judgment, and the potential for subjective bias in rulings. “*Due to the lack of official referees, there is a tendency for errors and disputes to arise during matches. As players, we may sometimes disagree due to differing perspectives.*” (P12). “*Some rules of the trust-based system may be somewhat ambiguous, leading to inconsistency in their enforcement. This can easily affect the fairness of the competition and our overall tournament experience.*” (P19).

Participants’ reports explain that the emergence of the tennis trust-based system has, to some extent, resolved controversies in amateur tournaments, but they have also raised some objections and expressed concerns about the trust-based system. By continuously refining and adjusting the trust-based system, we can gradually reduce these issues and enhance the overall fairness and enjoyment of the matches.

#### 3.2.3. Monitor and Detect Stage

We define the “monitor and detect stage” as the phase where players in tennis matches observe their opponents’ strokes, referee gestures, and the court environment. This stage precedes the implementation of the tennis trust-based system and involves players gathering anticipatory information about officiating decisions. Monitor refers to the monitoring of the entire court environment, while detect focuses on observing the action of the ball touching the ground.

When applying the trust-based system, players rely on their own experience in sports practice to judge the landing point of the tennis ball. “*During the match, I pay attention to observing the surrounding environment, especially conditions like wind speed and lighting, which directly affect my judgment of the ball’s landing point.*” (P10). Especially in non-professional matches without referees, participants are very concerned about whether the ball is out of bounds, particularly regarding the trajectory of the ball. “*In our matches, referees are often absent, so we have to rely on our own observations to quickly judge the ball’s trajectory to maintain fairness in the game.*” (P19). The monitor and detect stage explains how trust-based tennis rules affect players’ judgment during implementation. It elucidates how players rely on self-monitoring and detection in matches to uphold fairness and the spirit of competition.

#### 3.2.4. Anticipate and Respond Stage

The “anticipate and respond stage” occurs during the implementation of the trust-based tennis rules, where players anticipate the tennis ball about to land on the court and react to whether the ball stays within the boundaries, revealing the interactive nature of the trust-based tennis rules.

In this study, amateur tennis enthusiasts reported on their responses and feedback during the implementation of trust-based rules, including the psychological pressures and stress, controversies over critical judgment moments, the role of players’ ethical standards, and the acceptance or questioning of opponents’ judgment outcomes. “*Indeed, in trust-based tennis, disputes can arise at times. Without referees present, we rely on our own eyes and integrity to judge whether the ball is out or in the valid area. In such situations, disagreements may occur due to differing perspectives, especially at crucial moments determining the outcome.*” (P20). If trust-based systems are not applied effectively, they can also have adverse effects on the players. “*This system also brings us quite a bit of psychological fluctuations. We tend to favor rulings in our own favor. Sometimes, even though the ball clearly landed within the court boundaries, opponents claim it’s out. It really affects my mood, and it’s frustrating. I hope there will be more auxiliary technologies to solve this problem.*” (P13).

Additionally, at this stage, the impact of the trust-based system is more reflected in the interaction between the players. At this point, mutual trust, values, and familiarity levels become crucial, and the acceptance of each other’s rulings will directly reflect this interaction. “*In this trust-based tennis competition, I prefer to compete with friends whom I know rather than trusting strangers. After all, people tend to have their own interests, especially when dealing with someone unfamiliar.*” (P7). A more critical point is that existing amateur competitions, especially youth tournaments, may involve interests and honors, which can make such rules more biased. “*I have previously served as a referee in youth tournaments and have encountered some disputes among players, especially in qualification and group stages where there are no referees. Under the trust-based rules, everyone tends to favor themselves, even arguing over each point with their opponents because it involves their own interests. This is an inevitable phenomenon in these rules.*” (P5).

In tennis matches, it is understandable that both players seek their own interests, leading to occasional disputes. In the anticipate and respond stage of tennis matches, participants continuously engage in communication and negotiate the outcomes of rulings with their opponents. While participants express a desire for friendly exchanges and hope for better technology to assist in making rulings, the trust-based system persists in the long term due to cost considerations and other factors. Thus, interactive feedback and communication between both sides remain inevitable in tennis competitions.

#### 3.2.5. Development Improvement Stage

The trust-based system in tennis has a significant impact and challenge on the fairness of the game. This system emphasizes self-monitoring and integrity among players, motivating them to maintain high levels of focus and responsibility during matches, ensuring that every decision aligns with the rules of the competition. However, the lack of formal referees can lead to biases and disputes in judgment, especially in crucial moments. Therefore, players need to possess excellent judgment skills and maintain a calm demeanor to address these challenges, ensuring the fairness of the game and upholding the spirit of fair competition.

We focused on a professional tennis referee, aiming to understand the development trends of the tennis trust-based system through his years of adjudication experience. “*There is indeed room for improvement in the rules of the tennis trust-based system. To enhance its fairness and operational clarity, clearer and more specific game rules and standards could be established, especially regarding criteria for determining whether a ball is out of bounds. Additionally, systematic participant training and education can improve players’ judgment and discipline. Utilizing technological tools such as video replays can reduce errors and disputes. Importantly, fostering a positive competitive culture and community atmosphere that emphasizes integrity and fair play will encourage players to conscientiously adhere to the trust-based system. These improvement measures can enhance the tennis trust-based system, making it more robust and sustainable, thereby providing better support for the conduct of matches and the experience of participants.*” (P5, National Tennis umpire). Some participants also expressed concerns, “*In tense score situations, opponents often tend to favor their own judgments, which is truly infuriating.*” (P14).

The trust-based system fundamentally relies on human judgment, transitioning from third-party referees to mutual adjudication between the players themselves, inevitably leading to some controversy. Both professional referees and amateur tennis enthusiasts have voiced concerns about this rule. Moving forward, improvements to the trust-based system rules will need to address ethical, psychological, and educational aspects.

## 4. Discussion

### 4.1. Implications for Theory

The ‘trust-based system’, derived from a culture of mutual respect, equality, and sportsmanship in tennis, evoked deep admiration. In these rules, players completely delegated the responsibility of determining whether their shots were in or out to their opponents, maintaining ‘absolute trust’ in them. This practice not only distinguished tennis from other sports but also highlighted its unique emphasis on gentlemanly conduct. The trust-based system was not just a ‘code of conduct’; it also carried an expectation that compared to winning or losing, ‘sportsmanship’ and ‘integrity’ were more significant. This study conducted interviews and analyzed focus group discussions with 23 amateur tennis enthusiasts to explore their understanding and opinions on the tennis trust-based system rules. Participants included university professors, professional tennis referees, government officials, and sports teachers, reflecting on the operational mechanisms of the trust-based system in various amateur settings.

Firstly, based on the grounded theory, we analyzed four key factors of the tennis trust-based system rules: interest-driven, information acquisition and judgment, interactive communication, and development strategy. The open-ended results of grounded theory demonstrated that interest-driven factors have facilitated the development of amateur tennis, especially in economically developed regions. Tennis, once seen as an exclusive sport, has become more accessible due to its simplicity and health benefits. This accessibility has made tennis attractive and encourages competition across different ages, genders, and skill levels, transforming it into a comprehensive sport [46]. As amateur tennis competitions continue to evolve, the trust-based system is gradually taking the lead [24]. This required participants to exert greater effort, akin to the information acquisition and judgment categories outlined by grounded theory. They need to pay closer attention to the court environment to effectively implement this system and ensure fairness. Despite this, the trust-based system can reduce the cost of organizing competitions and improve efficiency [28]. However, the core of the trust-based system remains the interaction between the players themselves, representing a human-centric adjudication rule similar to referee judgments. Due to various human biases and factors, human-based judgments are prone to errors [47]. Additionally, the vulnerability of the trust system can lead to difficulty in decision-making for participants in complex situations. When faced with high levels of uncertainty or emotional pressure, participants may exhibit biases in their trust judgments, which can affect their behavior and decision-making. In a competitive environment, particularly for players with a strong desire to win, relying on a trust-based system for officiating presented a psychological challenge. Such a system required players to remain calm during tense moments and respect their opponents’ judgments, which had varying effects on players from different backgrounds. Experienced players, especially those with considerable competitive experience, had higher expectations regarding the fairness of the officiating system, making them more likely to doubt or distrust it during critical moments. This psychological state weakened their adherence to the trust-based system, especially when decisions went against them; they were more inclined to question their opponents’ judgments, potentially leading to disputes. In contrast, less experienced amateur players might have been more inclined to adhere to the trust-based system. For them, maintaining trust and sportsmanship was often more important in their competition experiences, and the pressure of winning was relatively lower. As a result, these players were generally more accepting of their opponents’ judgments, even if there were occasional misjudgments. This differing emphasis on competition could have significantly influenced how players perceived and committed to the trust-based system.

Secondly, based on the coding results of the grounded theory, we adapted the basic framework of PCM, constructing a model of the operational mechanisms of the tennis trust-based system. This model aimed to capture the environmental conditions of specific scenarios and suggests how participants can interpret them to inform their actions and decisions [48]. We divided the tennis trust-based system into five stages. The first stage was the foundational stage, where amateur tennis enthusiasts joined the sport due to interests, hobbies, leisure, and other means. The next stage was the emergence of the trust-based system, where the increasing number of participants and objective conditions facilitated the effective resolution of issues through this system. Following that was the monitoring and detection stage. During matches, participants needed to continuously adjust their relationship with the external environment before applying these rules to ensure proper implementation and fairness. Furthermore, there was the anticipation and response stage. During the operation of the trust-based system, constant communication between both participants was necessary. Differences of opinion between them were inevitable at this stage, leading to disputes that required careful attention. Lastly, there was the development and improvement stage. Every rule had its limitations, and the fairness of the trust-based system fundamentally depended on human judgment. It was essential to continuously improve the regulatory aspects of the tennis trust-based system from ethical and other human-centered perspectives [49].

The model of the operational mechanisms of the tennis trust-based system, based on grounded theory research, captures the social context in which behaviors occur [50]. By mapping this model onto the PCM framework, our approach centered on amateur tennis participants, effectively addressing concerns regarding human factors in the tennis trust-based system. It explained the theoretical framework of trust in amateur tennis competitions and laid a foundation for further enhancing the trust-based system.

### 4.2. Recommendations

The operational mechanism model of the tennis trust-based system we proposed explains the application scenarios and interaction processes within tennis competitions. However, we also identified some concerns from participants regarding this system. To address these concerns, we offer the following policy development suggestions:

Firstly, establish an integrity management system. The effective implementation of the tennis trust-based system in competition mode is closely related to the ethical foundation of participating players [51]. Therefore, sports integrity is crucial. Establishing an integrity management system that includes strict screening and assessment of participating players is essential to ensure fair competition. For example, implementing a credit rating mechanism to rank and score players and imposing penalties or bans on players who are dishonest or make erroneous judgments can enhance players’ awareness of integrity. Additionally, establishing a disciplinary notice board and setting up channels for reporting and appeals can enhance player self-discipline and oversight mechanisms, ensuring that matches are conducted in a fair and honest manner. In the selection and review of players, strict selection criteria should be established and publicly disclosed so that both players and spectators can understand and accept them, thereby enhancing the transparency and credibility of the competition.

Secondly, establish unified standards and processes. Event organizers should ensure that competition rules are easily understood and implemented through standardized rule training and process design. This includes detailed rule training courses, standardized processes, establishment of rule interpretation mechanisms, and ongoing management supervision to enhance the fairness of the competition and participant satisfaction. For example, establish oversight teams such as event supervision and data analytics groups, clearly defining responsibilities and divisions of labor. These teams would be collectively responsible for managing and guiding the trust-based system in tennis competitions, overseeing and managing the entire competition process. Additionally, there should be strengthened education and training for participants, which is crucial to ensuring the effective implementation of the rules [52].

### 4.3. Limitations

The study reveals the tensions and conflicts that arise with the application of tennis trust-based system rules. Despite these contributions, it also has its limitations. The study population selected for this paper, although comprising 23 tennis players from all walks of life, is not representative of all tennis participants’ perceptions of the trust-based system and may need to consider expanding the sample in the future. Testing the trust-based system in more competitive or professional environments is essential. Research could consider conducting studies at different levels of competition (such as youth, adult, or professional leagues) to further validate the effectiveness and adaptability of the trust system. In addition, the construction of the trust theory system model is based on self-reporting, which is somewhat subjective, and in this case, not everyone may truthfully report their true thoughts, which is a reflection of social expectations that cannot be completely avoided [53]. Future research could adopt a broader range of quantitative methods, such as surveys, to encompass a larger population of amateur tennis participants, ensuring the representativeness of the results. Additionally, using mixed methods (combining qualitative and quantitative approaches) could provide a more comprehensive perspective. For instance, alongside interviews, incorporating observational methods could offer deeper insights into the operation of the trust system in actual matches. Furthermore, long-term tracking studies (longitudinal research) could help us understand the dynamic processes of how the trust system evolves over time.

## 5. Conclusions

The study proposes a human-centered trust theory framework model based on grounded theory. Through focus group discussions and interviews and using a three-stage coding process, it identifies 28 main factors influencing the rules of the tennis trust-based system. These factors are categorized into four major groups: interest orientation, information acquisition and judgment, interactive communication, and development strategies. Furthermore, building upon this foundation, a PCM framework was used to construct a model of the operational mechanisms of the tennis trust-based system. This model encompasses five stages: foundation stage, trust-based emergence stage, monitor and detect stage, anticipate and respond stage, and development improvement stage.

In the context of introducing more technologically assisted officiating systems (such as Hawk-Eye and VAR), the establishment of a trust-based system provides a new perspective on interpersonal interactions and self-refereeing in competitions. In the absence of technological support, a trust system can operate effectively; however, it requires standardized management mechanisms and processes to reduce disputes and enhance fairness. By implementing clear guidelines and protocols, players can engage in a more collaborative and respectful environment, which reinforces the principles of trust and sportsmanship, ultimately contributing to a smoother and more equitable competitive experience. Within the operational process of the trust system, the anticipate and respond stage is the most likely to generate controversy. To address the shortcomings of the trust system, we can establish integrity management systems and standardized processes. This study effectively explains the formation mechanism and process of the tennis trust-based system, providing a theoretical foundation for the development of amateur tennis trust-based competitions. Future research can further explore how trust-based systems can adapt to other amateur sports events and various participant groups. By establishing new rule mechanisms for more amateur sports, such studies could promote fair competition and enhance officiating efficiency. Additionally, this exploration can provide new directions for future research on technology and trust systems, highlighting the importance of fostering trust and collaboration in diverse sporting contexts. Such investigations could lead to a deeper understanding of how these systems function across different environments, ultimately contributing to the development of more effective and equitable officiating practices in amateur sports.

## Figures and Tables

**Table 1:** Summary statistics of demographic variables.

Variables	N	%
Gender		
Male	18	78.3
Female	5	21.7
Age		
18–30	2	8.7
31–40	6	26.1
41–50	10	43.5
50–65	5	21.7
Tennis experience		
3–5 years	2	8.7
6–10 years	15	65.2
10–20 years	6	26.1
Occupation		
Professor	6	26.1
PE teacher	5	21.7
Administrative agent	3	13.1
National tennis umpire	1	4.3
Others	8	34.8

**Table 2:** Semi-structured interview outline.

No.	Question
1	How did you get into the sport of tennis?
2	Do you think you’re playing tennis to get something?
3	Have you ever played in an amateur tennis tournament? What are some of the things that matter to you during a tournament?
4	Have there been any disputes with opponents or referees during a match? What was the reason for each dispute, and did it affect your pace of play?
5	Tell us your thoughts or knowledge about the Tennis Trust-based System.
6	What do you think of rules like the Tennis Trust-based System?
7	Under the rules of the Tennis Trust-based System, describe your entire playing experience, including movements and mental swings.
8	Any other suggestions for the future of the Tennis Trust-based System?

**Table 3:** The three-stage coding results for grounded theory.

Open Coding	Axial Coding	Selective Coding
Like to watch professional tennis matches	Recreational hobbies	Interest-driven
Passion for tennis		
Enjoy the competition	Competitive entertainment	
Gain a sense of accomplishment		
Monitoring of the playing field	Objective information	Information acquisition and judgment
Observe where the tennis ball lands		
Observe the opponent’s movements		
Observe the referee’s hand signals	Subjective information	
Subjective judgment of where the ball lands		
Communicating penalties with opponents	Feedback and acceptance	Interactive communication
Negotiation of the disputed ball		
Acceptance of opponent’s penalty		
Communication and interpretation of penalties		
Strengthening trust among players		
Dissatisfaction with or challenge of penalties		
Increased interaction between players		
Eliminates subjective misjudgments by referees		
Athletic ethics	Psychology and stress	
Psychological factors (tension, stress)		
Penalties at key moments of the game		
Reducing the cost of competition	Dominance	Development strategy
Improving the efficiency of the game		
Promotion and popularization of tennis		
Favorable to exploring innovative approaches		
Lack of credibility and seriousness	Technology standard	
Lack of harmonized implementation standards		
Lack of technical support		
Unclear rules		
Easy to misjudge		

## Data Availability

The data that support the findings of this study are available from the corresponding author upon reasonable request.
